# Spontaneous rupture of the urinary bladder misdiagnosed as gastrointestinal perforation: a diagnostic pitfall in post-radiation patients

**DOI:** 10.1093/jscr/rjaf425

**Published:** 2025-06-16

**Authors:** Shunya Kiriyama, Kei Takahashi, Masao Niwa, Seishiro Sekino, Masatoshi Hayashi

**Affiliations:** Japanese Red Cross Gifu Hospital, Department of Surgery, 3-36, Iwakura, Gifu, Gifu Prefecture 5028511, Japan; Japanese Red Cross Gifu Hospital, Department of Surgery, 3-36, Iwakura, Gifu, Gifu Prefecture 5028511, Japan; Japanese Red Cross Gifu Hospital, Department of Surgery, 3-36, Iwakura, Gifu, Gifu Prefecture 5028511, Japan; Japanese Red Cross Gifu Hospital, Department of Surgery, 3-36, Iwakura, Gifu, Gifu Prefecture 5028511, Japan; Japanese Red Cross Gifu Hospital, Department of Surgery, 3-36, Iwakura, Gifu, Gifu Prefecture 5028511, Japan

**Keywords:** bladder rupture, gastrointestinal perforation, radiation-induced injury

## Abstract

Bladder rupture is a rare but potentially life-threatening condition that can mimic gastrointestinal perforation, particularly when intra-abdominal free air is present. The case of an 82-year-old woman with a history of pelvic radiotherapy for cervical cancer that developed intra-abdominal free air after urinary catheterization is reported. The patient exhibited only mild abdominal tenderness, and computed tomography showed intra-abdominal free air. Emergency laparotomy was performed based on the radiological findings, showing intraperitoneal bladder rupture with the catheter extruded through the bladder wall. Retrospective analysis suggested that the combination of radiation-induced bladder wall fragility and mechanical irritation by the catheter contributed to the rupture. Clinicians should recognize that intra-abdominal free air is not always indicative of gastrointestinal perforation. Bladder rupture should be considered, particularly following radiation therapy in lower abdominal cancer patients. Detailed history taking and physical examination can help prevent misdiagnosis and unnecessary surgical intervention in cases with mild abdominal symptoms.

## Introduction

Bladder rupture is an uncommon but potentially life-threatening condition that can be classified as traumatic or non-traumatic. Traumatic cases are typically associated with blunt or penetrating injuries, whereas non-traumatic, or spontaneous, bladder rupture occurs in the absence of direct trauma and is far rarer. Known risk factors for spontaneous rupture include chronic urinary retention, alcohol intoxication, malignancy, and prior pelvic radiotherapy [1, 2]. Of these, radiation therapy is a recognized cause of delayed bladder wall fragility due to fibrosis and decreased tissue compliance [1]. In such patients, the clinical presentation may be subtle, and radiological findings such as intra-abdominal free air can easily be misinterpreted as gastrointestinal perforation. A case of intraperitoneal bladder rupture following urinary catheterization in a patient with a history of pelvic irradiation is presented.

## Case report

**Figure 1:**
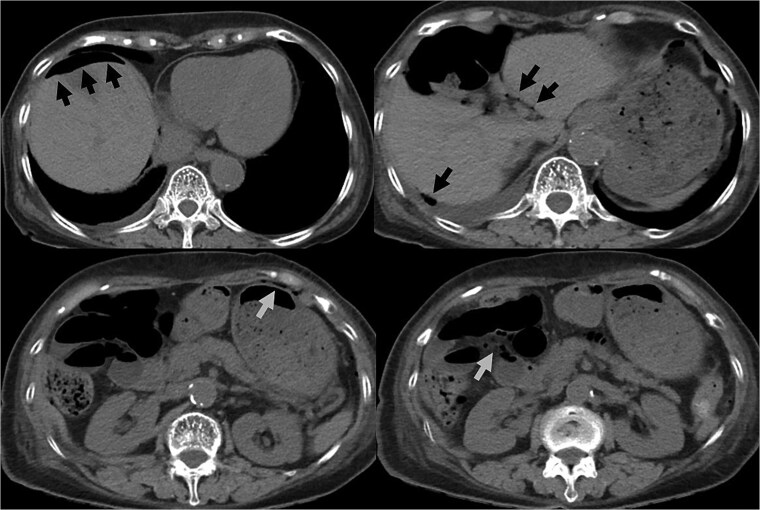
CT indicated intra-abdominal free air (arrows). These findings were later confirmed as being due to bladder rupture.

**Figure 2:**
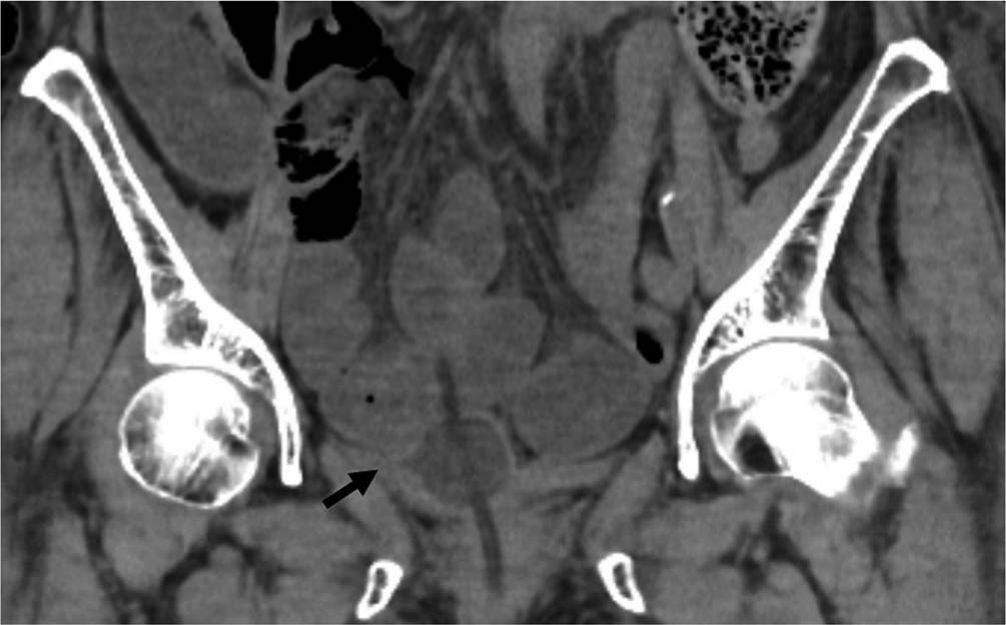
Coronal CT showed the urinary catheter was mispositioned outside the bladder (arrow).

**Figure 3:**
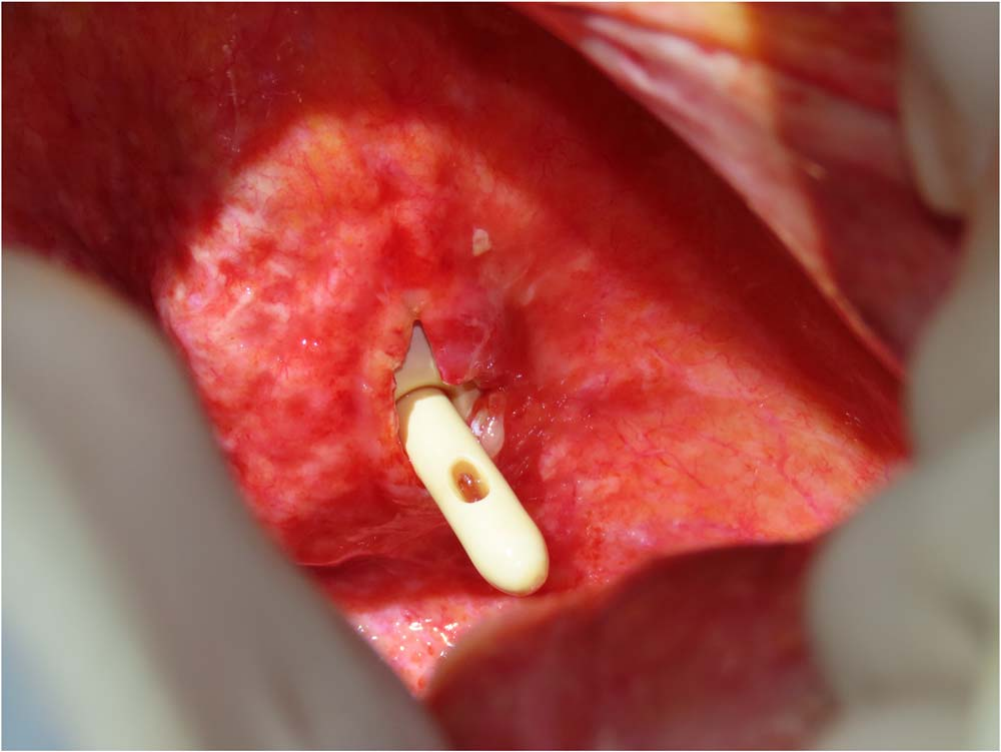
Intraoperative findings reveal bladder rupture with the urinary catheter protruding through the bladder wall. The bladder wall appears thin and fragile, consistent with radiation-induced damage.

An 82-year-old female with a history of cervical carcinoma presented to our hospital with lower abdominal pain. She had undergone a total hysterectomy decades ago for cervical carcinoma, followed by radiotherapy to the pelvis. Computed tomography (CT) revealed small bowel obstruction attributable to adhesions. The patient was initially diagnosed with adhesion-related small bowel obstruction and was managed conservatively with fasting and intravenous fluid replacement. Although her bowel function promptly recovered within a few days, urinary retention of unknown etiology was noted during hospitalization, necessitating clean intermittent catheterization. When this approach proved insufficient for relieving her symptoms, an indwelling urinary bladder catheter was inserted. Two days after insertion of the catheter, a follow-up abdominal X-ray was performed to confirm recovery of her bowel movement. Imaging revealed indwelling intra-abdominal air above the liver, which was confirmed by emergency computed tomography, raising the suspicion of gastrointestinal perforation (Figs 1 and 2). Clinically, her vital signs were stable, with a heart rate of 65 beats per minute, blood pressure of 113/72 mmHg, and body temperature of 36.6°C, and her abdomen was soft and flat with slight tenderness in the lower abdomen. Laboratory evaluation indicated a C-reactive protein level of 2.31 mg/dl and leukocytes of 5100/mm^3^. Emergency laparotomy was performed under general anesthesia to rule out potentially life-threatening conditions, such as gastrointestinal perforation. Intra-operatively, perforation of the urinary bladder was identified, with the urinary catheter protruding through the defect (Fig. 3). The bladder wall was extremely thin and fragile, suggesting significant loss of compliance. The bladder rupture was repaired using a two-layer closure with absorbable sutures, and the urinary catheter was repositioned to ensure proper drainage. The patient’s postoperative recovery was uneventful, and she was discharged on postoperative day 25 with the urinary catheter in situ. She was followed up for 70 days, and no signs of intra-abdominal leakage were observed on cystography. The catheter was removed without further complications.

## Discussion

Spontaneous rupture of the urinary bladder is an exceedingly rare condition. It is often difficult to diagnose due to its nonspecific symptoms, which can mimic more common conditions such as gastrointestinal perforation. The classical presentation typically includes lower abdominal pain [1]; however, some cases remain asymptomatic until the development of significant complications.

The etiology of spontaneous bladder rupture is multifactorial, with pelvic irradiation, alcohol intoxication, and post-vaginal delivery being the most frequently reported risk factors [1, 2]. Radiation therapy, in particular, is known to cause bladder rupture [3–5]. Chronic damage to the bladder wall leads to radiation cystitis [2], which induces inflammatory infiltration, fibrosis, and necrosis of the tissue [1]. In this case, the combination of radiation-induced bladder wall fragility and mechanical irritation from clean intermittent catheterization likely contributed to the rupture.

The diagnosis of bladder rupture can be particularly challenging in cases with atypical presentations. In this patient, the presence of intra-abdominal air initially led to the suspicion of gastrointestinal perforation, although the mild abdominal tenderness was atypical for this condition. Retrospective analysis revealed that the urinary catheter had been mispositioned outside the bladder after penetrating the bladder wall, further complicating the clinical picture. This highlights the importance of considering bladder rupture in the differential diagnosis of patients with a history of pelvic irradiation presenting with intra-abdominal air and nonspecific symptoms.

Management of spontaneous bladder rupture should be tailored to the individual patient’s condition [5]. Surgical repair is the preferred approach for cases involving significant leakage, peritonitis, or other life-threatening complications. Conversely, conservative management with bladder drainage might be appropriate in selected cases without severe infection or hemodynamic instability [1]. If the patient’s general condition permits, laparoscopic surgery may also be considered as a minimally invasive treatment option [6].

Bladder rupture should be considered in the differential diagnosis of intra-abdominal free air, particularly in patients with a history of pelvic irradiation. This case underscores the importance of careful history-taking and physical examination to prevent unnecessary surgical intervention for presumed gastrointestinal perforation.
